# Enhanced pulmonary uptake on ^18^F-FES-PET/CT scans after irradiation of the thoracic area: related to fibrosis?

**DOI:** 10.1186/s13550-019-0549-y

**Published:** 2019-08-23

**Authors:** C. M. Venema, E. F. J. de Vries, S. J. van der Veen, M. D. Dorrius, M. van Kruchten, C. P. Schröder, G. A. P. Hospers, A. W. J. M. Glaudemans

**Affiliations:** 1 Department of Medical Oncology, University of Groningen, University Medical Center Groningen, Groningen, the Netherlands; 2 Medical Imaging Center, Department of Nuclear Medicine and Molecular Imaging, University of Groningen, University Medical Center Groningen, Hanzeplein 1, 9700 RB Groningen, the Netherlands; 3 Department of Radiation Oncology, University of Groningen, University Medical Center Groningen, Groningen, the Netherlands; 4 Medical Imaging Center, Department of Radiology, University of Groningen, University Medical Center Groningen, Groningen, the Netherlands

**Keywords:** FES-PET, Estrogen receptor, Radiation therapy, Pulmonary fibrosis

## Abstract

**Rationale:**

The use of 16α-[^18^F]fluoro-17β-estradiol (FES) positron emission tomography (PET) in clinical dilemmas and for therapy decision-making in lesions expressing estrogen receptors is growing. However, on a considerable number of FES PET scans, previously performed in a research and clinical setting in our institution, FES uptake was noticed in the lungs without an oncologic substrate. We hypothesized that this uptake was related to pulmonary fibrosis as a result of radiation therapy. This descriptive study therefore aimed to investigate whether radiation therapy in the thoracic area is possibly related to enhanced pulmonary, non-tumor FES uptake.

**Methods:**

All FES-PET/CT scans performed in our institution from 2008 to 2017 were retrospectively analyzed. Scans from patients who had received irradiation in the thoracic area prior to the scan were compared to scans of patients who had never received irradiation in the thoracic area. The primary outcome was the presence of enhanced non-tumor FES uptake in the lungs, defined as visually increased FES uptake in the absence of an oncologic substrate on the concordant (contrast-enhanced) CT scan. All CT scans were evaluated for the presence of fibrosis or oncologic substrates.

**Results:**

A total of 108 scans were analyzed: 70 scans of patients with previous irradiation in the thoracic area and 38 of patients without. Enhanced non-tumor FES uptake in the lungs was observed in 39/70 irradiated patients (56%), versus in 9/38 (24%) of non-irradiated patients. Fibrosis was present in 37 of the 48 patients with enhanced non-tumor FES uptake (77%), versus in 15 out of 60 (25%) patients without enhanced non-tumor uptake, irrespective of radiotherapy (*p* < 0.001).

**Conclusion:**

After irradiation of the thorax, enhanced non-tumor uptake on FES-PET can be observed in the radiation field in a significant proportion of patients. This seems to be related to fibrosis. When observing enhanced FES uptake in the lungs, this should not be interpreted as metastases. Information on recent radiation therapy or history of pulmonary fibrosis should therefore be taken into consideration.

## Introduction

**T**he estrogen receptor (ER) is an important target for endocrine treatment in breast cancer patients, and ER expression of the tumor is the main indication to start antihormonal treatment, as success rates heavily rely on ER status [1, 2]. Although specificity and sensitivity of immunohistochemistry to assess ER expression are high, it is not always feasible to obtain a suitable biopsy. Moreover, ER expression can change over time in the metastatic setting and vary between the primary tumor and its metastases and between metastases within a single patient [3].

Non-invasive molecular imaging of the ER using positron emission tomography (PET) with 16α-[^18^F]fluoro-17β-estradiol (FES) has been found useful to detect the estrogen receptor status of the primary tumor and its metastases. FES-PET has been used in several imaging studies in breast cancer patients to visualize all metastases in a patient to assess tumor heterogeneity [4–13]. FES-PET has a high predictive value with a sensitivity of 85% and a specificity of 98% [14]. The uptake of FES differs per tissue type and anatomic site and can be influenced by intrinsic (i.e., menopausal status) and extrinsic factors (i.e., hormone therapy) [6, 9]. Recently, recommendations for the use of FES-PET, including the indications, correct patient preparation, scan acquisition, and analysis of the scans, were published [15].

The use of FES-PET in daily clinical practice, in patients with clinical dilemmas and for the detection of lesions expressing ER as input for treatment decisions, is growing. Therefore, it is important to gain more insight in the potential pitfalls that are associated with this imaging technique. In the University Medical Center Groningen, extensive experience is available with FES-PET scans in both a research and clinical setting [10, 11, 14, 15]. In a considerable number of FES-PET scans, heterogeneous uptake in the lungs was noticed, without the presence of an oncologic substrate on the accompanying (contrast-enhanced) CT scan. As this enhanced uptake was seen in the lungs and most patients were irradiated in the thoracic area, we hypothesized that pulmonary fibrosis as a result of earlier radiation therapy might be the cause of this FES uptake.

Since radiation therapy is one of the most frequently administered treatments in patients with breast cancer, and FES-PET is performed more and more in daily clinical practice, it is important for the interpretation of the scans to assess whether radiotherapy leads to enhanced FES uptake in the lungs.

Therefore, the aim of this descriptive study was to evaluate whether radiation therapy in the thoracic area is possibly related to enhanced pulmonary, non-tumor FES uptake.

## Methods

In this descriptive, single-center study, we retrospectively analyzed all FES-PET/CT scans that were performed for clinical purposes in our institution from 2008 to 2017. Information on irradiation was retrieved from the patient charts. Scans from patients who had received irradiation in the thoracic area prior to the scan were compared to scans of patients who had never received irradiation in the thoracic area. The medical history and radiation therapy schemes were retrieved from the electronic patient files. Given the retrospective descriptive nature of this study, national legislation does not require medical ethical approval; however, the local database registering patient objection has been checked for patients objecting against using their material.

### FES-PET

FES was produced in the University Medical Center Groningen by a two-step method that was extensively described previously [13]. In short, ^18^F-fluoride is prepared with a cyclotron by irradiation of ^18^O-water according to the nuclear reaction ^18^O(p,n)^18^F. The cyclotron-produced ^18^F-fluoride is allowed to react with 3-O-methoxymethyl-16,17-O-sulfuryl-16-epiestriol (ABX, Germany), followed by removal of the MOM protecting group and the sulfate group by hydrolysis with hydrochloric acid. After HPLC purification, the product is formulated in 10% ethanol in saline and sterilized by filtration [13]. FES with > 99% radiochemical purity was obtained in a practical yield of 3.4 ± 1.5 GBq. FES had a specific activity of 325 ± 274 GBq/μmol. Approximately 200 MBq of FES was injected intravenously. Whole body emission scans were performed approximately 60 min after tracer injection. PET images were obtained from skull base to mid-thigh with a Siemens 40 or 64 slice mCT (PET/CT) Biograph camera system (Siemens Medical Systems, Knoxville, TN, USA). A low-dose CT scan was performed in all patients for attenuation correction. Attenuation-corrected images were visually analyzed for enhanced non-tumor uptake. To calculate the uptake, a volume of interest (VOI) was drawn over the area of enhanced non-tumor uptake and the maximum standardized uptake value (SUV_max_) and the average SUV (SUV_mean_) using a 50% isocontour of the hottest pixel were measured using syngo.via software. In patients without visual enhanced non-tumor uptake, a VOI was drawn centrally in the lung (including the basal parts) for the same measurements. All scan acquisitions and calculations were performed according to EANM/EARL guidelines for ^18^F imaging [16].

### CT scan

All patients had a low-dose CT scan for attenuation correction at the time of FES PET. Part of the patients also had a contrast-enhanced CT scan within 6 weeks of the FES PET of the thorax available when this was clinically indicated. All CT scans were evaluated for fibrosis, or oncological substrates, by an experienced radiologist (*MD*). There are many features that may imply pulmonary fibrosis, such as honeycombing, traction bronchiectasis, lung architectural distortion, and reticulation. In case of radiation-induced pulmonary fibrosis, also other features may occur such as volume loss, linear scarring, chronic consolidation, mediastinal shift, and pleural thickening. All scans were checked for these features.

### Radiation schedules and dose

The patients who were irradiated received variable radiation schedules, depending on the indication and available techniques at the time of radiation therapy. To analyze the effect of different radiation doses and schemes on enhanced non-tumor FES uptake, FES-PET scans were fused with the original radiation therapy planning CT scans, including the radiation fields and doses, using Raystation software. Radiation doses were determined by drawing a VOI in the radiation field in the area with enhanced FES uptake. In patients that were irradiated but did not show enhanced uptake in the lungs, a VOI was drawn in the lungs, in the same region as for the SUV calculation.

### Statistics

The main outcome was the presence of enhanced non-tumor FES uptake, defined as visually increased FES uptake above background in the absence of an oncologic substrate on the concordant (low-dose or contrast-enhanced) CT scan. Correlations between enhanced FES uptake and radiation dose and between interval time between radiation therapy and FES PET scan were calculated using a Pearson correlation test. One-way ANOVA was used to analyze the statistical significance between group differences. A probability (*p*) value < 0.05 was considered statistically significant.

## Results

### Demographic data

In total, 133 scans were evaluated of which 108 scans were included for the analysis. FES-PET/CT scans were either performed for clinical dilemma patients with metastatic disease of unknown primary or to gain more insight in ER expression of metastases.

**Fig. 1:**
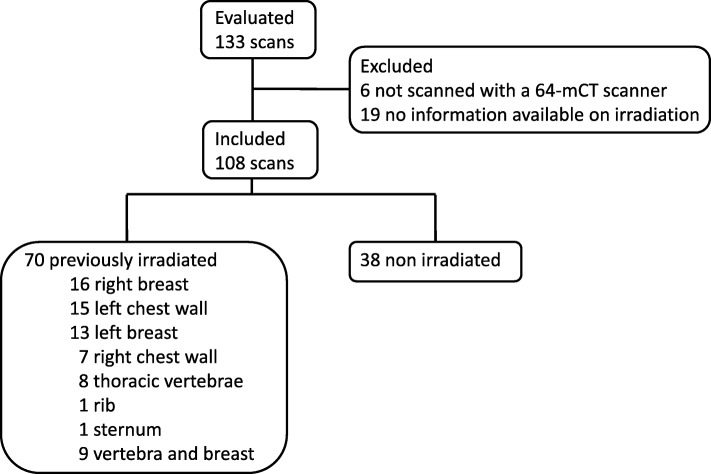
Consort diagram of included FES PET scans

In total, 70 patients were previously irradiated (Fig. 1). The majority of patients had breast cancer (98%); the other patients had either prostate cancer (1%) or ovarian cancer (1%). Mean age at the time of FES-PET scan was 59 years (range 33–86 years). The control group consisted of 38 patients. Detailed patient characteristics are described in Tables 1 and 2.

**Table 1:** Patient characteristics

	No radiation therapy (*n* = 38)	Radiation therapy (*n* = 70)	Overall (*n* = 108)
Age (mean, range)	57 (41–77)	60 (33–86)	59 (33–86)
Type of cancer			
Breast	36	70	106
Prostate	1	0	1
Endometroid	1	0	1
Treatment at time of PET scan			
None	15	31	46
Aromatase inhibitor	14	29	43
Chemotherapy	5	5	10
Estrogen degrader	1	3	4
Other	3	2	5

**Table 2:** Cross table for patient distribution based on radiation therapy and FES PET and the presence of visually enhanced uptake on FES PET

	Normal uptake	Enhanced uptake	Total
No radiation therapy	29	9	38
Radiation therapy prior to FES PET	31	39	70
Total	60	48	108

### More non-tumor FES uptake on FES-PET scans from irradiated patients

**Fig. 2:**
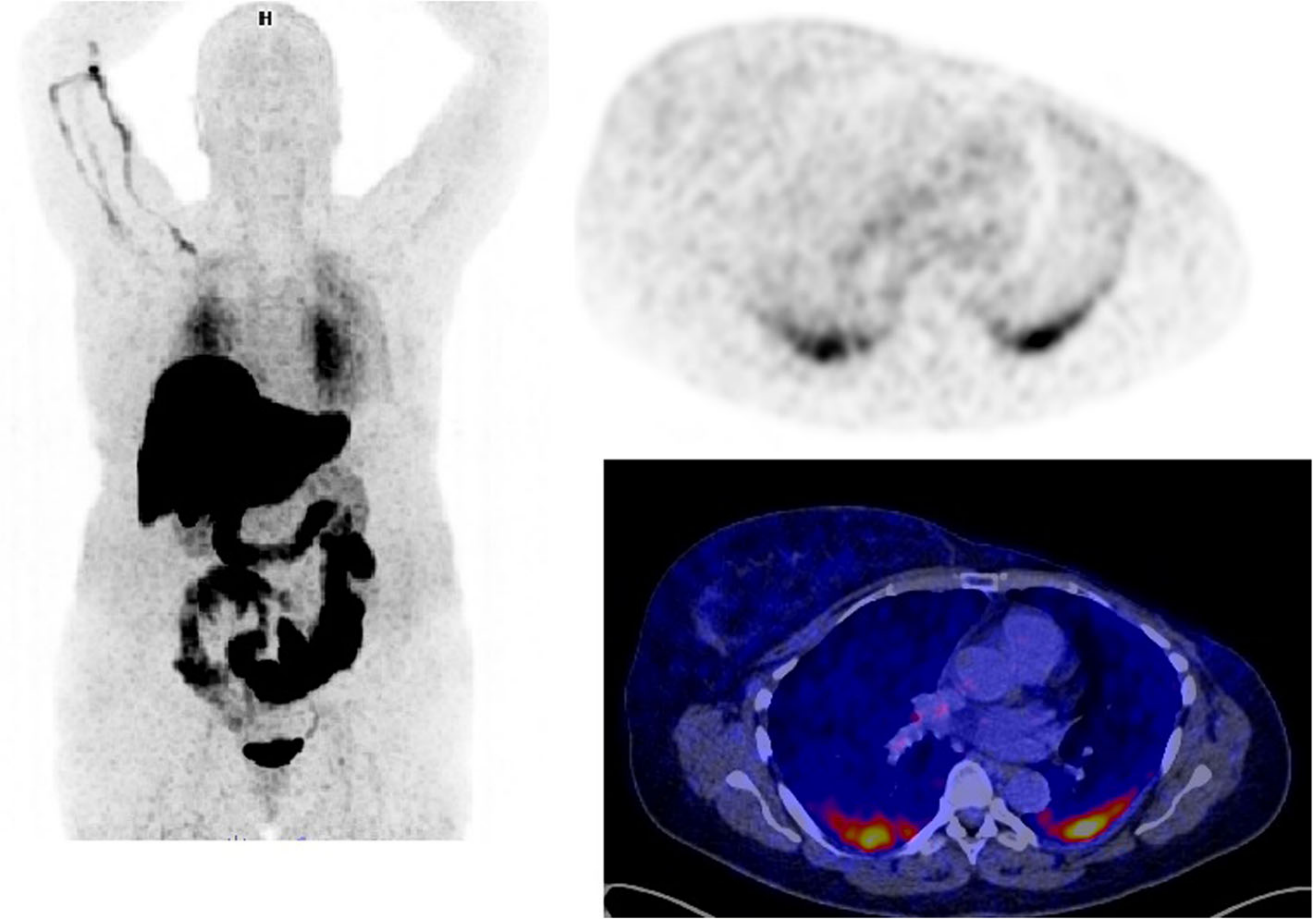
An example of a FES-PET/CT scan with high non-tumor uptake in the lungs

On 48/108 scans (44%), enhanced FES uptake in the lungs was observed without the presence of an oncological substrate on CT (Fig. 2 for an example). Enhanced non-tumor uptake was mostly located at the dorsomedial side of the lungs, and on 23/48 (48%) scans bilateral enhanced non-tumor uptake was seen. Quantitative PET analysis confirmed that tracer uptake was significantly higher in the patients with visually increased non-tumor FES uptake, compared to patients without nonspecific enhanced uptake (SUV_max_ 2.5 [SD 1.3] versus 1.0 [SD 0.2]; *p* < 0.001 and SUV_mean_ 1.5 [SD 0.8] versus 0.7 [SD 0.2]; *p* < 0.001). Enhanced non-tumor FES uptake in the lungs was observed in 39/70 scans in irradiated patients (56%), versus 9/38 scans of non-irradiated patients (23%) (Tables 3 and 4).

**Table 3:** Visually enhanced non-tumor uptake in the lungs on the FES PET scan and the presence of fibrosis on the concurrent CT scan in patients previously treated with radiation therapy of the thorax

	Normal uptake	Enhanced uptake	Total
No fibrosis	21	9	30
Fibrosis	10	30	40
Total	31	39	70

**Table 4:** Visually enhanced non-tumor FES uptake in the lung on the FES PET scan and the presence of fibrosis on concurrent CT scan in patients without any prior radiation therapy of the thorax

	Normal uptake	Enhanced uptake	Total
No fibrosis	24	2	26
Fibrosis	5	7	12
Total	29	9	38

### More fibrosis in patients with enhanced non-tumor FES uptake

In 66/108 patients, also contrast-enhanced CT scans of the thorax were available. For all other patients, low-dose CT scan for attenuation correction was available. Fibrosis was present in 52 patients (48%), of which 37 were diagnosed on a contrast-enhanced CT scan, and 15 on a low-dose CT.

Fibrosis was present in 77% of the patients with enhanced non-tumor FES uptake (37/48), versus 25% of the patients without enhanced non-tumor uptake (15/60, *p* < 0.001), irrespective of radiotherapy. Those patients with fibrosis without irradiation (12/38) were known with interstitial lung disease (*n* = 2), chronic obstructive pulmonary disease (*n* = 3), prior pneumothorax (*n* = 1), fibrotic string (*n* = 1), or unknown cause of fibrosis (*n* = 5).

### Enhanced non-tumor FES uptake is not related to the interval between irradiation and FES-PET, or radiation dose

**Fig. 3:**
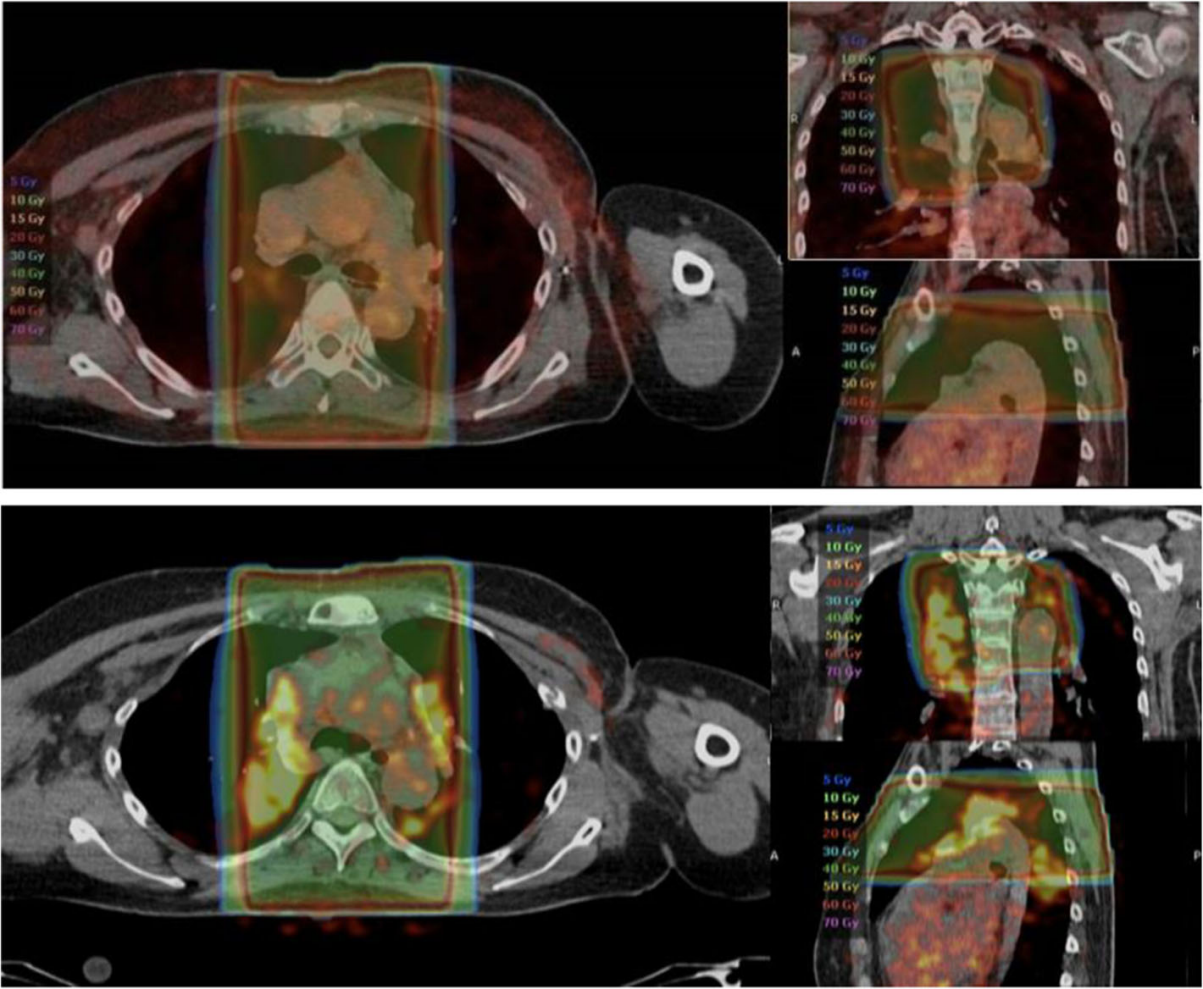
FES PET scan demonstrating normal uptake 3 weeks after radiation therapy of the mediastinal lymph nodes (upper panel). Two months after radiation therapy, the FES PET scan demonstrated enhanced non-tumor uptake (lower panel)

Of those patients that were previously irradiated (*n* = 70), the mean radiation dose was not different between patients without enhanced non-tumor FES uptake (*n* = 31, 95.9 Gy [SD 23.3]) versus patients with enhanced non-tumor FES uptake (*n* = 39, 89.1 Gy [SD 26.5]). No correlation could be found between SUV_max_ and radiation dose (*R*^2^ = 0.02, *p* = 0.21). The mean interval between the FES-PET scan and the last day of radiation therapy was 381 weeks (range 0–1450). There was no correlation between the time interval between FES-PET and radiation therapy, and SUV_max_ (*R*^2^ = 0.01, *p* = 0.43) or SUV_mean_ (*R*^2^ = 0.02 *p* = 0.14). However, in one patient, serial FES-PET scans were available on which enhanced non-tumor uptake was not present shortly after radiation therapy, but enhanced uptake was visual after several months (Fig. 3).

## Discussion

In the present study, we found enhanced non-tumor pulmonary FES uptake in a subset of patients, most frequently after radiation therapy in the thoracic area. Uptake of FES is considered to be ER specific, and the cause of this non-tumor uptake is not fully elucidated yet. However, this study supports a possible fibrosis-related origin. This aspect of non-tumor FES uptake on FES-PET has not been described before, and this is the largest series so far to allow hypothesis generation with regard to this aspect.

One possible cause of the enhanced tracer uptake is that the tracer binds to inflammation-related ERβ expression. Two isoforms of ER exist, α and β, and despite the fact that ^18^F-FES has a 6.3 times higher affinity for ERα compared to ERβ [12], uptake can be seen in ERβ-driven pathology [17]. Under normal conditions, low levels of ERβ are present in ovaries, the kidney, the brain, bone, the heart, the lungs, intestinal mucosa, the prostate, the immune system, and endothelial cells [18]. Also, in patients with interstitial pneumonia and cystic fibrosis, ERβ expression is higher than in healthy lung tissue [19, 20]. Both cystic fibrosis and interstitial pneumonia are marked by lung fibrosis and inflammation.

Both ERβ and ERα play a role in inflammation and fibrosis. Estrogen-dependent ERα activation is required for normal development of the dendritic cells [21] and high levels of dendritic cells are present in patients with lung fibrosis [22]. During inflammation, dendritic cells are activated to initiate and coordinate immune responses. We observed fibrosis or post-radiation inflammation in most patients with enhanced non-tumor FES uptake, but not in all. This could be explained by the timing of the CT scans. Fibrosis may not yet be detectable on a CT scan in an early stage of the formation of fibrosis. Exposure to radiation therapy could lead to side effects, largely depending on the anatomic site and dose received [23].

The pathogenesis of radiation-induced side effects is not fully understood but seems mostly related to extended inflammatory effects. As part of the inflammatory process, fibrosis may occur several weeks after radiation therapy [24]. The late phase typically occurs between 6 and 12 months and can continue to progress up to several years [25]. In 23 out of the 48 patients, enhanced uptake was seen bilaterally, which was beyond the boundaries of the radiation field. It has been reported, both preclinically and clinically, that bilateral radiation therapy toxicity may occur [26–29]. This suggests that enhanced FES uptake may be associated with a (late) inflammatory event caused by irradiation, also outside the irradiation field. Not in all patients, a contrast-enhanced CT scan was available, and due to the lower image quality of the low-dose CT, small areas of fibrosis could be missed.

Not all fibrosis in patients is related to radiation therapy. Extensive literature exists on lung toxicity due to several systemic treatments. With the wide time interval between irradiation and FES-PET treatment types, as well as treatment regimens and doses have changed over the years. With the retrospective design of the current study, we were unable to establish other correlations between fibrosis and FES uptake.

Another explanation for enhanced uptake in irradiated lungs is that radiation results into leakage of the blood vessels, possibly leading to extravasation of FES. In a preclinical rat model, radiation of the lungs showed vascular damage early after irradiation and remodeling leading to increased permeability, perivascular edema, and vascular remodeling [29, 30]. As a compensatory effect, the blood pressure, blood flow, and thereby shear stress may increase in the vasculature in the non-irradiated part of the lungs. This increase of shear stress may then lead to damage to the non-irradiated vasculature [30] and potentially explain leakage of the tracer in surrounding tissue. Though unbound FES can readily permeate the endothelium, most FES is bound to the sex hormone-binding globulin (SHBG) which, in case of leaky vessels, may also leak out.

FES-PET scans are increasingly used, both in a research and a clinical setting. The scans are often qualitatively assessed and lesions are identified as ER-positive if the tracer uptake is above the background signal. Therefore, it is important for the analysis of the scans to know that non-tumor uptake in the lungs may occur and that this finding should not be interpreted as pathological uptake. Also, existing lesions in the radiation field may potentially be non-evaluable in cases where the background signal is increased due to the uptake after radiation treatment. Furthermore, to facilitate the interpretation of FES-PET scans, semi-quantitative analysis can be performed and correction for physiologic background uptake is often applied when calculating SUV using the unaffected contralateral site or surrounding tissue of the same origin. In such cases, one should keep in mind that background activity in the reference region can be influenced by radiation therapy and consequently background correction may cause an underestimation of the tracer uptake in the lesion.

Despite the limitations of being a retrospective study over a long period of time, this is the most comprehensive series of patients receiving FES PET scans after radiation therapy described so far. The clinical significance of these findings has to be further investigated, e.g., the relation between the lung function of the patients and enhanced uptake. These data were not available in our patient charts. As such, the findings described here should be regarded as hypothesis generating and should preferably be confirmed in larger, prospective studies.

## Conclusion

In a substantial number of FES-PET scans, heterogeneous uptake in the lungs was noticed, without the presence of an oncologic substrate. This non-tumor pulmonary uptake is most probably related to fibrosis or inflammation caused by earlier radiation therapy. For a correct interpretation of FES-PET scans, information on recent radiation therapy, or history of pulmonary fibrosis of any cause should therefore be taken into consideration and pulmonary uptake should not immediately be interpreted as being caused by metastases.

## Data Availability

The datasets used and/or analyzed during the current study are available from the corresponding author on reasonable request.

## Abbreviations

ER: estrogen receptor
FDG: 2-deoxy-2-[^18^F]fluoro-D-glucose
FES: 16α-[^18^F]fluoro-17β-estradiol
SUV: standardized uptake value

## References

[1] Cardoso F, Harbeck N, Fallowfield L, Kyriakides S, Senkus E, ESMO Guidelines Working Group (2012). Locally recurrent or metastatic breast cancer: ESMO clinical practice guidelines for diagnosis, treatment and follow-up. Ann Oncol.

[2] Allred DC, Harvey JM, Berardo M, Clark GM (1998). Prognostic and predictive factors in breast cancer by immunohistochemical analysis. Mod Pathol.

[3] Simmons C, Miller N, Geddie W (2009). Does confirmatory tumor biopsy alter the management of breast cancer patients with distant metastases?. Ann Oncol.

[4] Dehdashti F, Mortimer JE, Trinkaus K (2009). PET-based estradiol challenge as a predictive biomarker of response to endocrine therapy in women with estrogen-receptor-positive breast cancer. Breast Cancer Res Treat.

[5] Kurland BF, Peterson LM, Lee JH (2001). Between-patient and within-patient (site-to-site) variability in estrogen receptor binding, measured in vivo by 18F-fluoroestradiol PET. J Nucl Med.

[6] Linden HM, Kurland BF, Peterson LM (2011). Fluoroestradiol positron emission tomography reveals differences in pharmacodynamics of aromatase inhibitors, tamoxifen, and fulvestrant in patients with metastatic breast cancer. Clin Cancer Res.

[7] Linden HM, Stekhova SA, Link JM (2006). Quantitative fluoroestradiol positron emission tomography imaging predicts response to endocrine treatment in breast cancer. J Clin Oncol.

[8] Peterson LM, Kurland BF, Schubert EK (2014). A phase 2 study of 16α-[^18^F]-fluoro-17β-estradiol positron emission tomography (FES-PET) as a marker of hormone sensitivity in metastatic breast cancer (MBC). Mol Imaging Biol.

[9] Peterson LM, Kurland BF, Link JM (2011). Factors influencing the uptake of ^18^F-fluoroestradiol in patients with estrogen receptor positive breast cancer. Nucl Med Biol.

[10] van Kruchten M, de Vries EG, Glaudemans AW (2015). Measuring residual estrogen receptor availability during fulvestrant therapy in patients with metastatic breast cancer. Cancer Discov.

[11] van Kruchten M, Glaudemans AW, de Vries EF (2012). PET imaging of estrogen receptors as a diagnostic tool for breast cancer patients presenting with a clinical dilemma. J Nucl Med.

[12] Seimbille Y, Rousseau J, Benard F (2002). ^18^F-labeled difluoroestradiols: preparation and preclinical evaluation as estrogen receptor-binding radiopharmaceuticals. Steroids.

[13] Yoo J, Dence CS, Sharp TL, Katzenellenbogen JA, Welch MJ (2005). Synthesis of an estrogen receptor β-selective radioligand: 5-[^18^F]fluoro-(2R,3S)-2,3-bis(4-hydroxyphenyl) pentanenitrile and comparison of in vivo distribution with 16α-[^18^F]fluoro-17β-estradiol. J Med Chem.

[14] van Kruchten M, de Vries EG, Brown M (2013). PET imaging of oestrogen receptors in patients with breast cancer. Lancet Oncol.

[15] Venema CM, Apollonio G, Hospers GAP (2016). Recommendations and technical aspects of 16α-[^18^F]fluoro-17β-estradiol PET to image the estrogen receptor in vivo: the Groningen experience. Clin Nucl Med.

[16] Boellaard R, Delgado-Bolton R, Oyen WJ (2015). FDG PET/CT: EANM procedure guidelines for tumour imaging: version 2.0. Eur J Nucl met. Mol Imaging.

[17] van Kruchten M, de Vries EF, Arts HJ (2015). Assessment of estrogen receptor expression in epithelial ovarian cancer patients using 16α-[^18^F]fluoro-17β-estradiol PET/CT. J Nucl Med.

[18] Couse JF, Lindzey J, Grandien K, Gustafsson JA, Korach KS (1997). Tissue distribution and quantitative analysis of estrogen receptor-α (ERα) and estrogen receptor-β (ERβ) messenger ribonucleic acid in the wild-type and ERα-knockout mouse. Endocrinology.

[19] Chotirmall SJ, Greene MC, Oglesby IK (2010). 17β estradiol inhibits IL-8 in cystic fibrosis by upregulating secretory leucoprotease inhibitor. Am J Respir Crit Care Med.

[20] Taniuchi S, Fujishima F, Miki Y (2014). Tissue concentrations of estrogens and aromatase immunolocalization in interstitial pneumonia of human lung. Mol Cell Endocrinol.

[21] Douin-Echinard V, Laffont S, Seillet C (2008). Estrogen receptor α, but not β, is required for optimal dendritic cell differentiation and CD40-induced cytokine production. J Immunol.

[22] Lammertyn EJ, Vandermeulen E, Bellon H (2017). End-stage cystic fibrosis lung disease is characterised by a diverse inflammatory pattern: an immunohistochemical analysis. Respir Res.

[23] Haviland JS, Owen JR, Dewar JA (2013). The UK standardisation of breast radiotherapy (START) trials of radiotherapy hypofractionation for treatment of early breast cancer: 10-years follow-up results of two randomised controlled trials. Lancet Oncol.

[24] Choi YW, Munden RF, Erasmus JJ (2004). Effects of radiation therapy on the lung: radiologic appearances and differential diagnosis. Radiographics.

[25] Barnett GC, West CM, Dunning AM (2009). Normal tissue reactions to radiotherapy: towards tailoring treatment dose by genotype. Nat Rev Cancer.

[26] Cohen Y, Gellei B, Robinson E (1974). Bilateral radiation pneumonitis after unilateral lung and mediastinal irradiation. Radiol Clin Biol.

[27] Martin C, Romero S, Sanchez-Paya J, Massuti B, Arriero JM, Hernandez L (1999). Bilateral lymphocytic alveolitis: a common reaction after unilateral thoracic irradiation. Eur Respir J.

[28] Coppes RP, Muijs CT, Faber H (2011). Volume-dependent expression of in-field and out-of-field effects in the proton-irradiated rat lung. Int J Radiat Oncol Biol Phys.

[29] van der Veen SJ, Faber H, Ghobadi G (2016). Decreasing irradiated rat lung volume changes dose-limiting toxicity from early to late effects. Int J Radiat Oncol Biol Phys.

[30] Ghobadi G, Bartelds B, van der Veen SJ (2012). Lung irradiation induces pulmonary vascular remodelling resembling pulmonary arterial hypertension. Thorax.

